# Circular RNA circPHKA2 Relieves OGD-Induced Human Brain Microvascular Endothelial Cell Injuries through Competitively Binding miR-574-5p to Modulate SOD2

**DOI:** 10.1155/2021/3823122

**Published:** 2021-11-08

**Authors:** Xiaobo Yang, Xiuying Li, Chuanhong Zhong, Jianhua Peng, Jinwei Pang, Tangming Peng, Weifeng Wan, Xianglong Li

**Affiliations:** ^1^Department of Neurosurgery, The Affiliated Hospital of Southwest Medical University, China; ^2^Sichuan Clinical Research Center for Neurosurgery, China; ^3^Academician (Expert) Workstation of Sichuan Province, China; ^4^Laboratory of Neurological Diseases and Brain Function, China; ^5^Department of Pharmacy, The Affiliated Hospital of Southwest Medical University, China

## Abstract

**Background:**

Circular RNA phosphorylase kinase regulatory subunit alpha 2 (circPHKA2; hsa_circ_0090002) has a significantly, specifically different expression in acute ischemic stroke (AIS) patients' blood. Here, we intended to investigate the role and mechanism of circPHKA2 in oxygen-glucose deprivation- (OGD-) induced stoke model in human brain microvascular endothelial cells (HBMEC).

**Methods:**

Expression of circPHKA2, microRNA- (miR-) 574-5p, and superoxide dismutase-2 (SOD2) was detected by quantitative PCR and western blotting. Cell injury was measured by detecting cell proliferation (EdU assay and CCK-8 assay), migration (transwell assay), neovascularization (tube formation assay), apoptosis (flow cytometry and western blotting), endoplasmic reticulum stress (western blotting), and oxidative stress (assay kits). Direct intermolecular interaction was determined by bioinformatics algorithms, dual-luciferase reporter assay, biotin-labelled miRNA capture, and argonaute 2 RNA immunoprecipitation.

**Results:**

circPHKA2 was downregulated in AIS patients' blood in SOD2-correlated manner. Reexpressing circPHKA2 rescued EdU incorporation, cell viability and migration, tube formation, B cell lymphoma-2 (Bcl-2) expression, and SOD activity of OGD-induced HBMEC and alleviate apoptotic rate and levels of Bcl-2-associated protein (Bax), glucose-regulated protein 78 kD (GRP78), C/EBP-homologous protein (CHOP), caspase-12, reactive oxygen species (ROS), and malondialdehyde (MDA). Additionally, blocking SOD2 partially attenuated these roles of circPHKA2 overexpression. Molecularly, circPHKA2 upregulated SOD2 expression via interacting with miR-574-5p, and miR-574-5p could target SOD2. Similarly, allied to neurovascular protection of circPHKA2 was the downregulation of miR-574-5p.

**Conclusion:**

circPHKA2 could protect HBMEC against OGD-induced cerebral stroke model via the miR-574-5p/SOD2 axis, suggesting circPHKA2 as a novel and promising candidate in ischemic brain injury.

## 1. Introduction

Acute ischemic stroke (AIS), accounting for approximately 85% of all strokes, occurs due to the obstruction of cerebral blood flow and the deficiency of oxygen and nutrients [1]. The China Stroke Prevention Project Committee (CSPPC) established in 2011 intends to promote stroke prevention and control and improve stroke care [2]. Pathophysiologically, cerebral ischemia causes a sequence of detrimental events, including inflammation, oxidative stress, endoplasmic reticulum (ER) stress, and apoptosis [3], as well as alteration of epigenetics [4, 5]. New pharmacological interventions, including noncoding RNA- (ncRNA-) based strategies, have shown promise for treatment of AIS.

Circular RNAs (circRNAs) are an evolutionarily conserved class of ncRNAs that are formed in a circular conformation via noncanonical splicing or backsplicing events [6]. Global expression of circRNAs is differentially altered in ischemic lesion tissue, peripheral blood, and peripheral blood mononuclear cell from AIS patients [4, 7, 8]. Circular RNAs are broadly involved in stroke-related neurovascular damages [9], and thus, circRNAs are proposed as novel diagnostic and prognostic biomarkers for AIS [7, 10]. Recently, circRNA phosphorylase kinase regulatory subunit alpha 2 (circPHKA2; hsa_circ_0090002) and circBBS2 (hsa_circ_0039457) are found to show significantly and specifically different expression in blood of AIS patients through the regulation of target genes in ischemic brain [10]. Existing literatures indicate that the two circRNAs seemed unstudied until now, and we chose circPHKA2 as one candidate for further functional analysis to decode the repertoires of circPHKA2 in IS-induced cerebral injury.

Superoxide dismutase-2 (SOD2) is a mitochondrial-specific antioxidant enzyme that specializes in scavenging oxygen free radicals and then eliminating oxidative stress [11]. In stroke, SOD2 genetic variant may alternatively predict the risk of stroke in patients with sickle cell anemia [12], and SOD2 mutation exacerbates ischemic brain damage under hyperglycemic condition [13]. SOD2 has been shown to be a vital enzyme in the maintenance of vascular function [14, 15], and inducing/activating SOD2 is a novel strategy for neurovascular protection after ischemia/reperfusion [16].

In this study, we attempted to explore the association between circPHKA2 and SOD2 in cerebral IS, and its cell model was established in human brain microvascular endothelial cells (HBMEC) induced by oxygen-glucose deprivation (OGD). Eventually, a circPHKA2-microRNA (miR-574-5p)-SOD2 axis was highlighted, and miR-574-5p is recommended as one of the markers for stroke diagnosis by interacting with multiple messenger RNAs (mRNAs) of stroke candidate genes [17].

## 2. Materials and Methods

### 2.1. Blood Samples and OGD-Induced Cerebral Stroke Cell Model

32 patients with AIS and 21 healthy people were selected in the Affiliated Hospital of Southwest Medical University. The venous blood from AIS patients was obtained before recanalization therapy, and control bloods were collected from subjects during physical examinations. The AIS patients were diagnosed according to the Guidelines for the Diagnosis and Treatment of AIS in China and MRI/CT results. Baseline characteristics of AIS patients and control subjects are shown in Table 1. This study was approved by the Ethical Committee of the Affiliated Hospital of Southwest Medical University.

Immortalized HBMEC were supplied by BNCC (BNCC337728, Beijing, China) and cultured in DMEM medium (Gibco, Rockville, MD, USA) supplemented with 5% fetal bovine serum (Gibco), 1% endothelial cell growth supplement (ECGS) (ScienCell), and 1x penicillin-streptomycin solution (Procell, Wuhan, China) in an incubator containing 95% sterile air/5% CO_2_ at 37°C. In the OGD model group, the medium was replaced with glucose-free DMEM for 6 h in a hypoxic incubator (Plas-Labs, Lansing, MI, USA) containing 95% N_2_/5% CO_2_. The control HBMEC were in normal medium harboring 4.5 g/L glucose under normal 95% sterile air/5% CO_2_ for 6 h. The cell supernatant and extract were collected after OGD for subsequent experiments.

### 2.2. Reverse Transcription PCR (RT-PCR) and Quantitative PCR (qPCR)

Total RNA was extracted from bloods, cells, and subcellular fractions using TRIzol LS reagent (Invitrogen, Carlsbad, CA, USA) according to the manufacturer's instructions. Nuclear/Cytosol Fractionation Kit (BioVision, San Francisco, CA, USA) was used to isolate the cytoplasm and nucleus of HBMEC. RT-PCR was performed to generate complementary DNA (cDNA) using RevertAid First Strand cDNA kit (Thermo Scientific, Waltham, MA, USA). Then, qPCR amplification was conducted using PowerTrack SYBR Green Master Mix (Thermo Scientific) and relevant primers for circPHKA2, PHKA2, miR-574-5p, SOD2, GAPDH, and U6. The primers are summarized in Table 2. The cycle threshold (Ct) values were obtained according to three biological duplications, normalized to the level of GAPDH or U6, and compared with the control group. RNA expression level was calculated using the 2^−△△Ct^ method [18].

### 2.3. Identification of Circular Structure and circRNA Stability

First-strand cDNA of HBMEC was synthesized from total RNA with random hexamer primer (for all RNAs) or oligo (dT)18 primer (for only RNAs containing poly (A) tails), which were provided in RevertAid First Strand cDNA kit (Thermo Scientific).

Genomic DNA (gDNA) from HBMEC was isolated using Mammalian Cell Genomic DNA Isolation Kit (BioVision), and circPHKA2 and GAPDH were amplified in cDNA and gDNA from HBMEC using divergent primers and convergent primers. And an aliquot of RT-PCR products was visualized by agarose gel electrophoresis, and other RT-PCR products were subjected to qPCR. By the way, the PCR product in divergent primers/cDNA group was confirmed by Sanger sequencing. The primers are summarized in Table 1.

4 U/*μ*g RNA RNase R (GENESEED, Guangzhou, China) was added into total RNA from HBMEC to digest linear RNA for 30 min at 37°C, and equal volume of 1x reaction buffer provided in this kit was added as the mock group. The resulting RNAs were subsequently purified using RNeasy MinElute Cleaning Kit (Qiagen) for RT-PCR and qPCR.

### 2.4. Cell Transfection

The pCD5-ciR vector (GENESEED) was used to overexpress circPHKA2, and empty vector was its negative control (NC). The micrON™ miR-574-5p and SOD2 siRNA (si-SOD2) were purchased from RIBOBIO (Guangzhou, China), as well as the negative controls miR-NC and si-NC. HBMEC and OGD-subjected HBMEC were administrated with these vectors/oligos using Lipofectamine 2000 (Invitrogen) as recommended by the manufacturer.

### 2.5. EdU Assay and Cell Counting Kit- (CCK-) 8 Assay

Cell-Light EdU DNA Cell Proliferation Kit (RIBOBIO) and CCK-8 (Dojindo, Kumamoto, Japan) were adopted to detect cell proliferation of HBMEC according to 5 × 10^4^ cells/well in a 96-well plate. Fluorescence density was analyzed under an inverted fluorescence microscope (ZEISS, Shanghai, China), and the percentage of EdU-positive (EdU+) cells was calculated normalized to DAPI+ cells. The absorption at 450 nm was examined using a microplate reader, and relative cell viability was determined comparing to a value of 100% in the control group.

### 2.6. Transwell Migration Assay

An 8 *μ*m pore transwell chamber (Becton-Dickinson, San Jose, CA, USA) and 24-well plate were together employed to measure cell migration. 5 × 10^5^ cells were inoculated in the upper chamber without serum and normal medium was in the lower chamber. After 48 h of incubation, cells that transferred onto the bottom face of the chamber were analyzed under a microscope following crystal violet staining. Migratory cells per field were counted according to five fields (at ×100) of interest.

### 2.7. Tube Formation Assay

50 *μ*L Growth Factor Reduced Matrigel (Becton-Dickinson) was planted onto 96-well plates at 37°C for 30 min, and 1 × 10^4^ cells were planted in each well for 3 days. Ten random fields of interest were selected for imaging, and tube formation rate was analyzed on Quantity One software (Bio-Rad, Hercules, CA, USA).

### 2.8. Flow Cytometry (FCM)

Cell death of HBMEC with and without OGD treatment was determined by an Annexin V Apoptosis Detection Kit FITC (eBioscience, San Diego, CA, USA) and a flow cytometer (Becton-Dickinson). Apoptosis analysis was performed using Cell Quest Research Software (Becton-Dickinson) by determining the percentage of early apoptotic cells and late apoptotic/necrotic cells [19].

### 2.9. Western Blotting

The western blot analysis was performed as previous study described [20] using 25 *μ*g total protein per lane. The primary antibodies (B cell lymphoma-2 (Bcl-2; A0208, 1 : 500, ABclonal), Bcl-2-associated X (Bax; A7626, 1 : 1000, ABclonal), glucose-regulated protein 78 kD (GRP78; A4908, 1 : 10,000, ABclonal), C/EBP-homologous protein (CHOP; A0854, 1 : 1000, ABclonal), caspase-12 (A0217, 1 : 1000, ABclonal), SOD2 (A19576, 1 : 1000, ABclonal), and *β*-actin (AC038, 1 : 50,000, ABclonal)) were used. HRP Goat Anti-Rabbit IgG (H+L) (AS014, 1 : 10,000, ABclonal) was used for secondary antibody. The protein bands were exposed by ChemiDoc™ XRS Imaging System (Bio-Rad), and band density was quantified using Quantity One software (Bio-Rad). *β*-Actin was used to correct levels of special proteins.

### 2.10. Assay Kits for Reactive Oxygen Species (ROS), Malondialdehyde (MDA), and SOD

SOD activity and levels of ROS and MDA were separately measured using commercially assay kits (Beyotime, Shanghai, China) for total SOD, ROS, and lipid peroxidation MDA. Cell lysates of HBMEC were prepared in cold PBS, and protein concentration was quantified using Enhanced BCA Protein Assay Kit (Beyotime, Shanghai, China). SOD activity and MDA level were calibrated by protein content. ROS level in HBMEC was evaluated by incubating in fluorescence probe DCFH-DA-containing serum culture medium, and fluorescence intensity of the transformed DCF was measured on fluorescence microplate at 488 nm excitation wavelength/530 nm emission wavelength.

### 2.11. Bioinformatics Algorithms and Dual-Luciferase Reporter Assay

CircInteractome, circBank, and starBase databases were used to predict the complementary sites of miRNAs in circPHKA2 and SOD2. The retrieval results of above databases were cross-screened using Venn diagram to find the overlapping miRNAs. These potential binding sites were examined using dual-luciferase reporter assay via pGL4 Luciferase Reporter Vectors (Promega, Madison, WI, USA).

To construct a report vector for wild-type (WT) of circPHKA2, gDNA from HBMEC was used to amplify the fragments (139-345 and 1519-1725) of circPHKA2 containing miR-574-5p response elements. The corresponding mutant-type (MUT) of circPHKA2 was generated by site-directed mutagenesis using Hieff Mut™ Site-Directed Mutagenesis Kit (YEASEN, Shanghai, China). Then, PCR products of WT-circPHKA2 and MUT-circPHKA2 were cloned into pGL4 vectors (Promega). Similarly, the fragment (308-517) of SOD2 3′UTR was chosen to generate WT-SOD3 3′UTR vector and MUT-SOD3 3′UTR vector. These recombinant vectors were cotransfected into HBMEC with commercial miR-574-5p or miR-NC for 48 h, and luciferase activities of luc2 (Photinus pyralis) and hRluc (Renilla reniformis) genes were examined using Dual-Glo® Luciferase Assay System (Promega) in differently transfected HBMEC.

### 2.12. Biotin-Labelled miRNA Capture and Argonaute 2 (Ago2) RNA Immunoprecipitation (RIP)

Biotin was used to label WT and MUT of miR-574-5p (WT/MUT-bio-miR-574-5p), which was provided by Genepharm (Shanghai, China), as well as the biotin-coupled miR-NC (bio-miR-NC). All of these bio-miRNAs were administrated in HBMEC for 48 h, and cell lysate was obtained for incubation in streptomycin-magnetic beads (NEB, England, UK). The pull-down RNAs were restored in TRIzol LS reagent (Invitrogen) for qPCR.

RIP-Assay Kit (MBL, Woburn, MA, USA) was used to perform IgG RIP in HBMEC using the normal rabbit IgG (negative control) or anti-IgG (RN003M, MBL). Protein A/G Agarose beads (YEASEN) were immobilized with 15 *μ*g antibodies at 4°C for 1 h with gentle rotation and then incubated in the precleared cell lysate of HBMEC at 4°C for 4 h. Then, antibody-immobilized bead-ribonucleoprotein (RNP) complex was used to restore total RNA as recommended by the manufacturer.

### 2.13. Statistical Analysis

GraphPad Prism7 (GraphPad, La Jolla, CA, USA) was used to analyze the results of our experiments, which were shown as means ± standard deviations (SD) of three independent samples. Difference comparisons were analyzed by Student's two-tailed *t*-test or one-way analysis of variance followed by the Bonferroni post hoc test. Statistical significance was accepted at *P* < 0.05.

## 3. Results

### 3.1. Exon-Derived circPHKA2 Was Decreased in AIS Patients and OGD-Induced HBMEC

**Figure 1:**
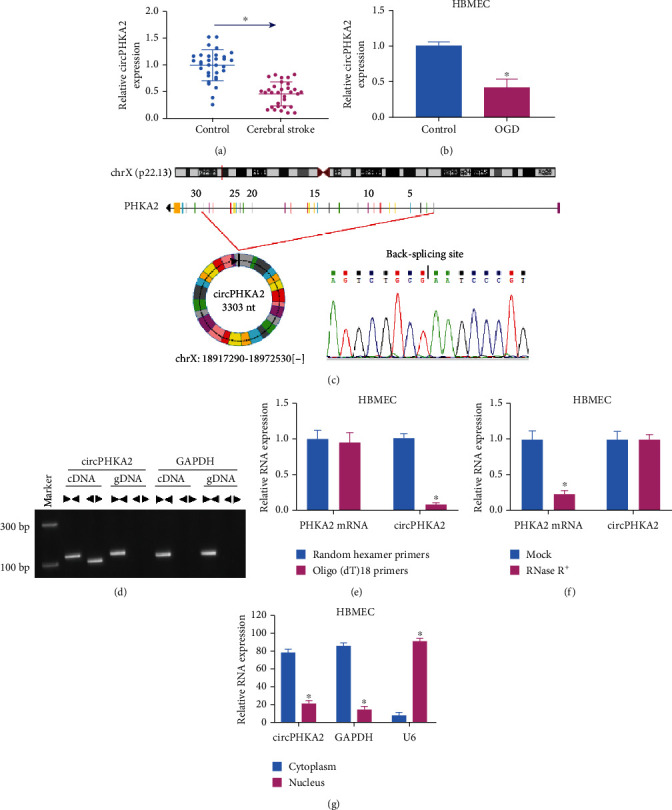
Exon-derived circPHKA2 was decreased in cerebral IS samples. (a, b) qPCR detected relative circPHKA2 expression in blood samples of AIS patients (*n* = 32) and healthy controls (*n* = 32) and cell samples of OGD-treated HBMEC and control HBMEC. (c) CircView software annotates the backsplicing of PHKA2 exons 2-29 according to the UCSC database, and Sanger sequencing confirmed the backsplicing site. (d) RT-PCR detected circPHKA2 and GAPDH levels using divergent primers and convergent primers in cDNA and gDNA from HBMEC. qPCR detected expression of circPHKA2 and PHKA2 mRNA in (e) HBMEC using random hexamer primers and oligo (dT)18 primers and in (f) RNase R-treated (RNase R+) and mock-treated total RNAs from HBMEC. (g) qPCR detected expression of circPHKA2, GAPDH, and U6 in the cytoplasm and nucleus of HBMEC. ^∗^*P* < 0.05.

According to this cohort of cerebral stroke patients, blood circPHKA2 expression was 0.46-fold of controls (Figure 1(a)). Besides, level of circPHKA2 was lower in HBMEC after OGD (Figure 1(b)). Thus, this circRNA was subjected to in-depth study. The UCSC database showed that circPHKA2 was produced from exons 2-29 of host gene PHKA2 located on chromosome X, and Sanger sequencing confirmed the backsplicing site of circPHKA2 (Figure 1(c)). Next, the circular structure of circPHKA2 was validated using special primers and RNase R treatment. Gel electrophoresis after RT-PCR indicated that circPHKA2 was only amplified by divergent primers in cDNA from HBMEC, while linear GAPDH was amplified by convergent primers in both cDNA and gDNA (Figure 1(d)); besides, linear PHKA2 mRNA could be amplified by random hexamer primers and oligo (dT)18 primers, whereas circPHKA2 expression was markedly decreased using the oligo (dT)18 primers (Figure 1(e)). On the other hand, PHKA2 mRNA expression was significantly descended after RNase R treatment, and circPHKA2 was resistant to that (Figure 1(f)). Furthermore, subcellular localization of circPPHKA2 was determined in nucleoplasmic separation, and the result was that circPHKA2, like GAPDH, was dominantly found in the cytoplasm (Figure 1(g)). These results indicated that circPHKA2 was a circular and stable transcript that was mainly located in the cytoplasm of HBMEC and was downregulated in AIS patients' blood.

### 3.2. circPHKA2 Prompted Proliferation, Migration, and Neovascularization of HBMEC after OGD

**Figure 2:**
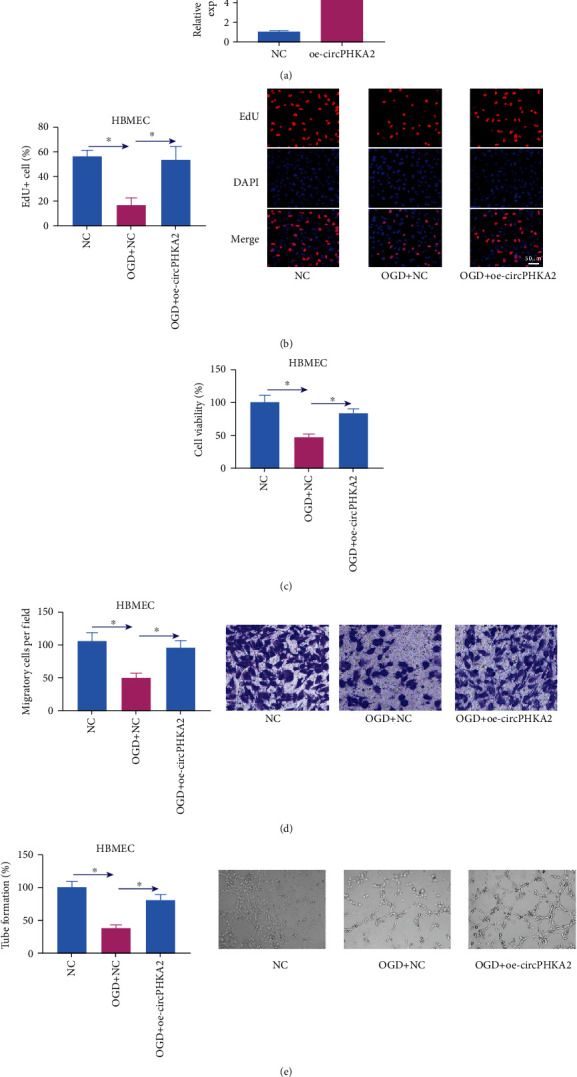
circPHKA2 prompted proliferation, migration, and neovascularization of HBMEC after OGD. (a) qPCR detected relative circPHKA2 expression in HBMEC administrated with oe-circPHKA2 or NC. (b–e) OGD-induced HBMEC were pretransfected with oe-circPHKA2 or NC, compared with control HBMEC transfected with NC. (b) EdU assay determined percentage of EdU+ cells. (c) CCK-8 assay measured percentage of cell viability. (d) Transwell assay confirmed number of migratory cells per field (×100). (e) Tube formation assay determined percentage of tube formation. ^∗^*P* < 0.05.

Gain-of-function experiments were carried out to measure role of circPHKA2 in dysfunction of HBMEC, and oe-circPHKA2 vector was used to overexpress circPHKA2 via transfection (Figure 2(a)). Cell proliferation of HBMEC was suppressed after OGD, as evidenced by the loss of EdU+ cells and cell viability (Figures 2(b) and 2(c)), whereas introducing oe-circPHKA2 vector could attenuate OGD-induced inhibition of cell proliferation (Figures 2(b) and 2(c)). Transwell assay depicted that OGD treatment decreased transwell migratory cells, and oe-circPHKA2-induced circPHKA2 overexpression could elevate migration of HBMEC (Figure 2(d)). Ectopic expression of circPHKA2 could also rescue neovascularization of HBMEC after OGD, as described by the improvement of tube formation rate in OGD-induced HBMEC with oe-circPHKA2 administration than NC administration (Figure 2(e)). These results demonstrated that reexpressing circPHKA2 might prompt proliferation, migration, and neovascularization of HBMEC after OGD.

### 3.3. circPHKA2 Inhibited Apoptosis, ER Stress, and Oxidative Stress of HBMEC after OGD

**Figure 3:**
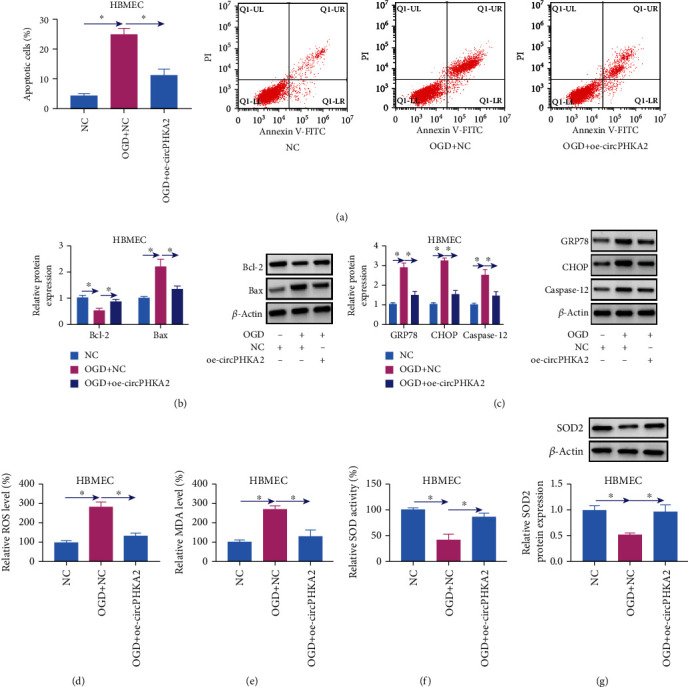
circPHKA2 inhibited apoptosis, ER stress, and oxidative stress of HBMEC after OGD. OGD-induced HBMEC were pretransfected with oe-circPHKA2 or NC, compared with control HBMEC transfected with NC. (a) FCM identified percentage of apoptotic cells. (b, c) Western blotting detected relative protein expression of Bcl-2, Bax, GRP78, CHOP, caspase-12, and *β*-actin. (d–f) Assay kits of ROS, MDA, and SOD examined relative ROS level, MDA level, and SOD activity, respectively. (g) Western blotting detected relative SOD2 protein expression, normalized to *β*-actin. ^∗^*P* < 0.05.

OGD induced high apoptosis in HBMEC (Figures 3(a) and 3(b)), and circPHKA2 overexpression via transfection diminished apoptotic cell rate of OGD-induced HBMEC accompanying with higher Bcl-2 and lower Bax (Figures 3(a) and 3(b)). Western blotting also showed the upregulation of ER-related proteins GRP78, CHOP, and caspase-12 in response to OGD treatment, and administrating oe-circPHKA2 could overall downregulate expression of those proteins (Figure 3(c)). Assay kits revealed that oxidative stress-associated factors ROS and MDA were highly induced whereas SOD activity was inhibited in OGD-induced HBMEC (Figures 3(d)–3(g)), which was salvaged with ectopic expression of circPHKA2 (Figures 3(d)–3(g)). These data demonstrated that reexpressing circPHKA2 might inhibit apoptosis, ER stress, and oxidative stress of HBMEC after OGD.

### 3.4. circPHKA2 Relieved OGD-Induced Injuries in HBMEC by Modulating SOD2

**Figure 4:**
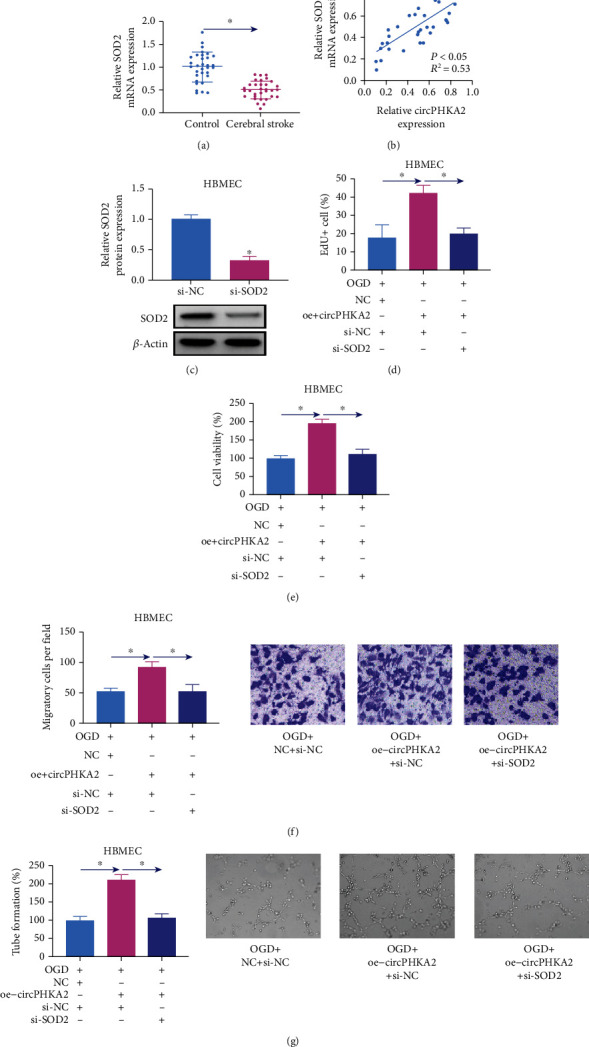
circPHKA2 prompted proliferation, migration, and neovascularization of HBMEC after OGD by modulating SOD2. (a) qPCR detected relative SOD2 mRNA expression in blood samples of AIS patients and healthy controls. (b) Pearson's correlation analysis analyzed the correlation between SOD2 mRNA and circPHKA2 expression in cerebral stroke patients. (c) qPCR detected relative SOD2 protein expression in HBMEC administrated with si-NC or si-SOD2. (d–g) OGD-induced HBMEC were pretransfected with oe-circPHKA2, NC, or oe-circPHKA2 combined with si-NC or si-SOD2. (d) EdU assay determined percentage of EdU+ cells. (e) CCK-8 assay measured percentage of cell viability. (f) Transwell assay confirmed number of migratory cells per field (×100). (g) Tube formation assay determined percentage of tube formation. ^∗^*P* < 0.05.

**Figure 5:**
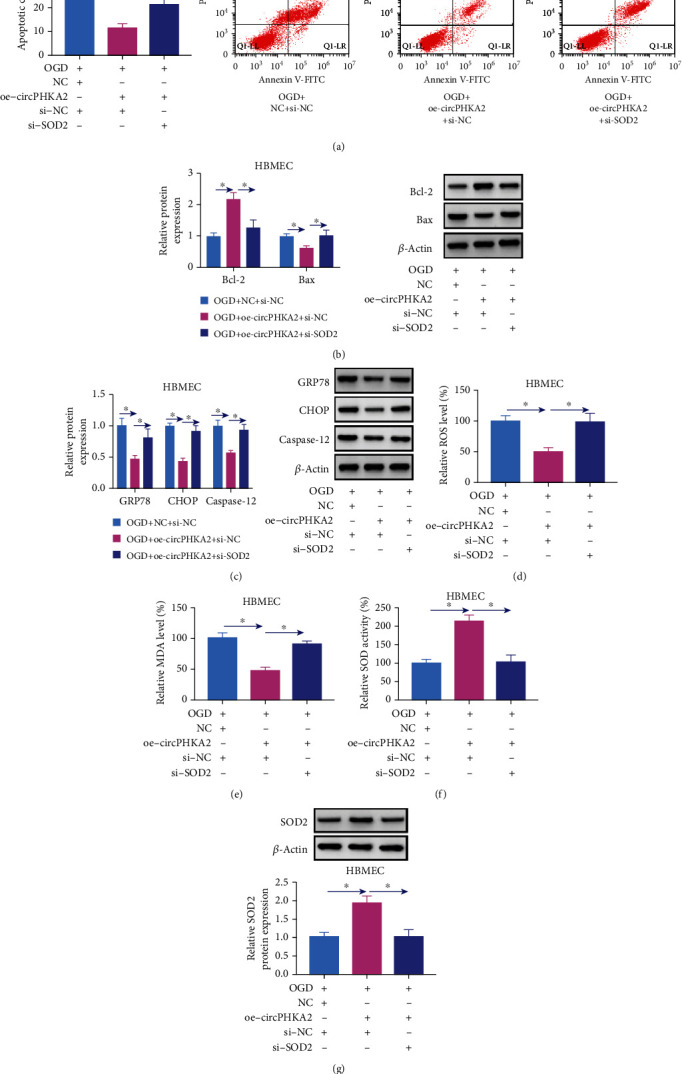
circPHKA2 inhibited apoptosis, ER stress, and oxidative stress of HBMEC after OGD by modulating SOD2. OGD-induced HBMEC were pretransfected with oe-circPHKA2, NC, or oe-circPHKA2 combined with si-NC or si-SOD2. (a) FCM identified percentage of apoptotic cells. (b, c) Western blotting detected relative protein expression of Bcl-2, Bax, GRP78, CHOP, caspase-12, and *β*-actin. (d–f) Assay kits of ROS, MDA, and SOD examined relative ROS level, MDA level, and SOD activity, respectively. (g) Western blotting detected relative SOD2 protein expression, normalized to *β*-actin. ^∗^*P* < 0.05.

We noticed that SOD2 mRNA expression was downregulated in the bloods from 32 AIS patients (Figure 4(a)), and this expression was positively and linearly correlated with circPHKA2 (Figure 4(b)). Functionally, SOD2 silencing in HBMEC via si-SOD2 administration could counteract the promoting roles of circPHKA2 in EdU incorporation and cell viability of OGD-induced HBMEC (Figures 4(c)–4(e)). Moreover, transwell migration and tube formation of HBMEC after OGD were rescued by overexpressing circPHKA2, and this rescue was weakened when si-SOD2 was introduced (Figures 4(f) and 4(g)). OGD-induced high apoptotic rate and Bax expression in HBMEC were decreased by upregulating circPHKA2, which were then increased by downregulating SOD2 (Figures 5(a) and 5(b)). Similarly, ER stress and oxidative stress of OGD-induced HBMEC were restrained by circPHKA2 reexpression, and unsurprisingly, this restraint was attenuated in the presence of si-SOD2, as indicated by the increased levels of GRP78, CHOP, caspase-12, ROS, and MDA (Figures 5(c)–5(e)), as well as decreased SOD2 level and SOD activity (Figures 5(f) and 5(g)). These results demonstrated that SOD2 inhibition partially abrogated the roles of circPHKA2 overexpression in OGD-induced HBMEC, suggesting that circPHKA2 might suppress OGD-induced injuries in HBMEC by modulating SOD2.

### 3.5. circPHKA2 Mediated the Regulation of SOD2 via Serving as a Sponge for miR-574-5p

**Figure 6:**
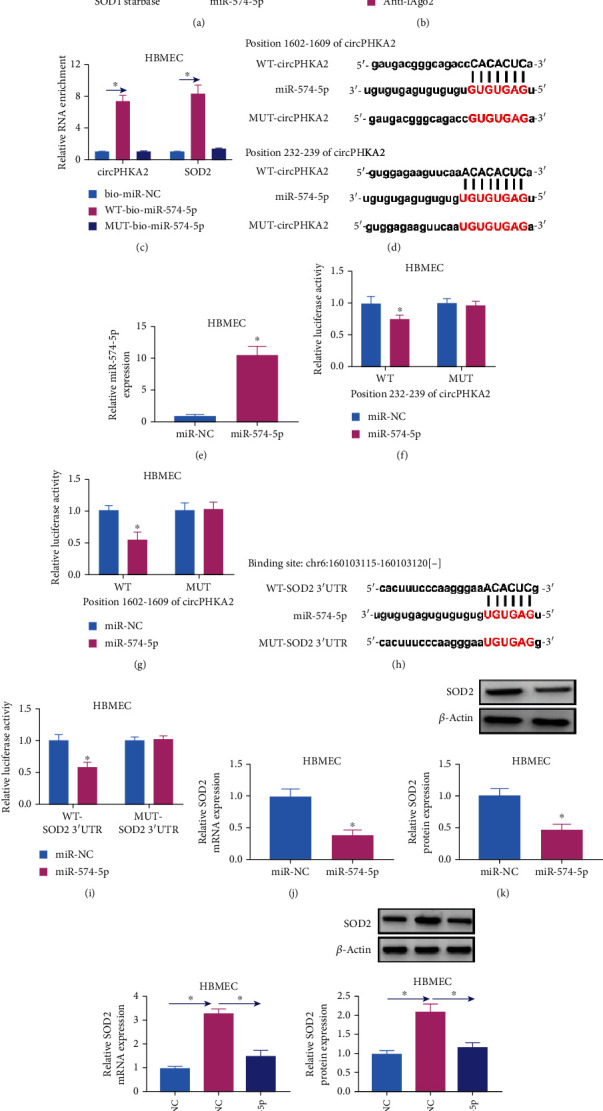
circPHKA2 mediated the regulation of SOD2 via serving as sponge for miR-574-5p. (a) Venn diagram showed the computational miRNAs that could target circPHKA2 and SOD2 according to CircInteractome, circBank, and starBase databases. (b) RIP and qPCR detected relative RNA enrichment of circPHKA2, SOD2, and miR-574-5p by anti-Ago2 in HBMEC, normalized to anti-IgG-mediated RIP. (c) Biotin-labelled miRNA capture examined relative RNA enrichment of circPHKA2 and SOD2 by probes of bio-miR-NC, WT-bio-miR-74-5p, and MUT-bio-miR-574-5p. (d) Schematic drawing showed the predicted binding sites between miR-574-5p and WT-circPHKA2 or MUT-circPHKA2 according to two positions of circPHKA2. (e) qPCR detected relative miR-574-5p expression in HBMEC administrated with commercial miR-574-5p or miR-NC. (f, g) Dual-luciferase reporter assay measured relative luciferase activity of WT-circPHKA2 vectors and MUT-circPHKA2 vectors in HBMEC administrated with commercial miR-574-5p or miR-NC. (h) Schematic drawing showed the predicted binding sites between miR-574-5p and WT-SOD2 3′UTR or MUT-SOD2 3′UTR. (i) Dual-luciferase reporter assay measured relative luciferase activity of WT-SOD2 3′UTR vector and MUT-SOD2 3′UTR vector in HBMEC administrated with commercial miR-574-5p or miR-NC. qPCR and western blotting examined relative SOD2 mRNA and protein expression in HBMEC administrated with (j, k) commercial miR-574-5p or miR-NC and (l, m) oe-circPHKA2 along with commercial miR-574-5p or miR-NC and NC along with miR-NC. ^∗^*P* < 0.05.

Furthermore, the link between circPHKA2 and SOD2 was further explored, and bioinformatics algorithms put forward that miR-574-5p was complementary to the primary sequences of both circPHKA2 and SOD2 (Figure 6(a)). Ago2 RIP assay showed a coenrichment of circPHKA2, SOD2, and miR-574-5p in cell lysate of HBMEC (Figure 6(b)), and RNA pull-down assay revealed that circPHKA2 and SOD2 were coenriched by WT-bio-miR-574-5p rather than MUT-bio-miR-574-5p (Figure 6(c)). According to CircInteractome, there were two predicted miR-574-5p-binding positions of circPHKA2, and the potential response elements were mutated (Figure 6(d)). Commercial miR-574-5p was administrated into HBMEC to overexpress miR-574-5p (Figure 6(e)), and this overexpression significantly reduced relative luciferase activities of WT-circPHKA2 report vectors, leaving alone the MUT vectors (Figures 6(f) and 6(g)). Likewise, starBase-predicted miR-574-5p response elements in SOD2 3′UTR were site-mutated (Figure 6(h)), and miR-574-5p transfection could descend the luciferase activity of WT-SOD2 3′UTR vector (Figure 6(i)). Ectopic expression of miR-574-5p and circPHKA2 could, respectively, downregulate and upregulate SOD2 expression levels (Figures 6(j)–6(m)); meanwhile, miR-574-5p overexpression was an obstacle for the regulatory effect of circPHKA2 on SOD2 (Figures 6(l) and 6(m)). These findings showed that there was a direct interaction between circPHKA2 and miR-574-5p and miR-574-5p and SOD2 and that circPHKA2 might modulate SOD2 expression via miR-574-5p.

### 3.6. circPHKA2 Relieved OGD-Induced Injuries in HBMEC by Controlling miR-574-5p

**Figure 7:**
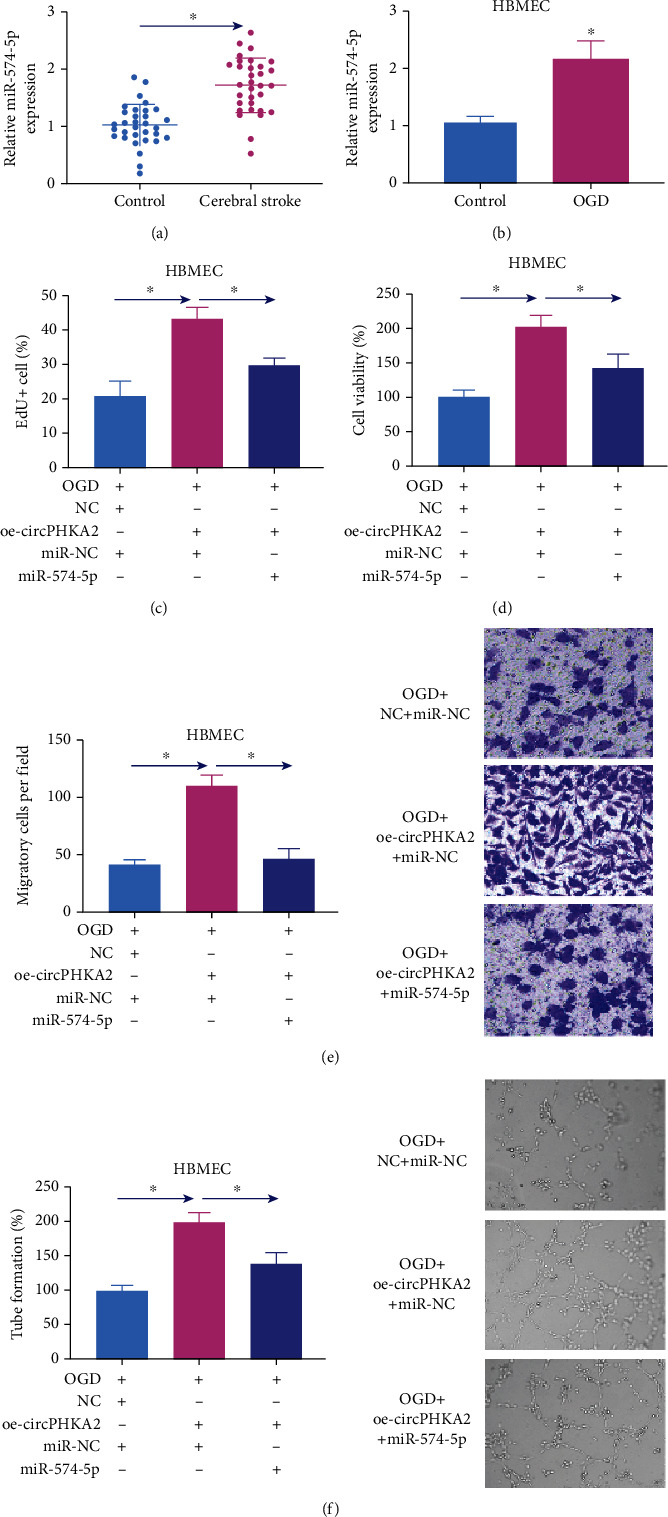
circPHKA2 regulated proliferation, migration, and neovascularization of HBMEC after OGD by controlling miR-574-5p. (a) qPCR detected relative miR-574-5p expression in blood samples of AIS patients and healthy controls. (b) qPCR detected relative miR-574-5p expression in control HBMEC and HBMEC treated with OGD. (c–f) OGD-induced HBMEC were pretransfected with oe-circPHKA2, NC, or oe-circPHKA2 combined with commercial miR-574-5p or miR-NC. (c) EdU assay determined percentage of EdU+ cells. (d) CCK-8 assay measured percentage of cell viability. (e) Transwell assay confirmed number of migratory cells per field (×100). (f) Tube formation assay determined percentage of tube formation. ^∗^*P* < 0.05.

**Figure 8:**
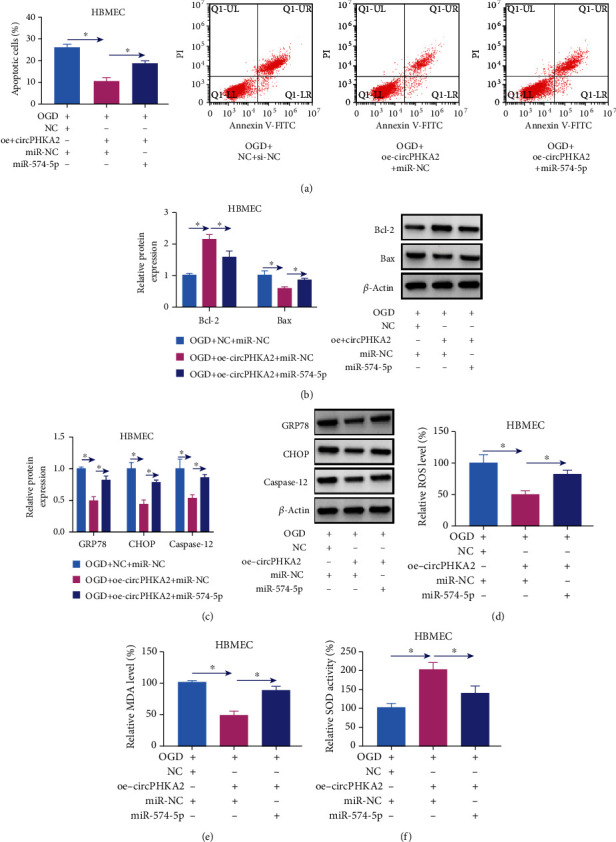
circPHKA2 inhibited apoptosis, ER stress, and oxidative stress of HBMEC after OGD by modulating SOD2. OGD-induced HBMEC were pretransfected with oe-circPHKA2, NC, or oe-circPHKA2 combined with commercial miR-574-5p or miR-NC. (a) FCM identified percentage of apoptotic cells. (b, c) Western blotting detected relative protein expression of Bcl-2, Bax, GRP78, CHOP, caspase-12, and *β*-actin. (d–f) Assay kits of ROS, MDA, and SOD examined relative ROS level, MDA level, and SOD activity, respectively. ^∗^*P* < 0.05.

Expression of miR-574-5p was 2.11-fold high in blood of AIS patients and OGD-treated HBMEC (Figures 7(a) and 7(b)). Cotransfecting with commercial miR-574-5p, oe-circPHKA2 vector-mediated promotions of EdU+ cells, cell viability, transwell migratory cells, and tube formation rate in HBMEC after OGD were partially abated (Figures 7(c)–7(f)), as well as the inhibitions of apoptotic cells and expression of Bax, GRP78, CHOP, and caspase-13, as well as productions of ROS and MDA (Figures 8(a)–8(e)). SOD2 activity was salvaged by ectopic circPHKA2 in OGD-induced HBMEC, and it was further declined due to commercial miR-574-5p (Figure 8(f)). These results concluded that circPHKA2 protected HBMEC from OGD-induced injuries by controlling miR-574-5p.

## 4. Discussion

IS was a multifactorial disease contributing to mortality and neurological dysfunction. Currently, circRNAs such as hsa_circ_0000607, circFUNDC1, circPDS5B, and circCDC14A had been promising candidates as biomarkers and targets in Chinese population-based AIS patients, due to their high specificity and stability [7, 21]. Here, we investigated the dysregulation of circulating circPHKA2 in AIS patients and further confirmed the role and competitive endogenous RNA (ceRNA) regulatory mechanism of circPHKA2 in neurovascular damage induced by OGD.

First of all, expression of circPHKA2 was observed to be lower in blood of AIS patients and cell model of cerebral IS induced by OGD in HBMEC. The downregulation of blood circPHKA2 in stroke patients was previously reported by Lu et al. [10]. Then, we characterized the circular structure and stability of circPHKA2 using a series of assays. As a result, Sanger sequencing identified the head-to-tail connection of exon-born circPHKA2, and circPHKA2 could not be amplified by convergent primers and oligo (dT)18 primers, suggesting that circPHKA2 showed no linear structure. Furthermore, circPHKA2 expression was found to be resistant to RNase R digestion, and this finding additionally indicated circPHKA2 with a loop conformation. Next, subcellular location assay indicated that circPHKA2 was inclined to be cytoplasmic distribution, which was consistent with the notion that exon-derived circRNAs were mostly located in the cytoplasm and functioned as miRNA sponges [22]. And subsequently, it was validated that circPHKA2 could sponge miR-574-5p to regulate SOD2 expression in HBMEC, and colocalization of the three RNAs was also confirmed by the RIP and pull-down assays. By the way, miR-1962 might be another target for circPHKA2 in regulating functional genes in the ischemic brain [10].

Functionally, allied to reexpression of circPHKA2 in OGD-induced HBMEC was the improvement of cell proliferation, migration, and neovascularization and the mitigation of apoptosis, oxidative stress, and ER stress. These findings indicated the neurovascular protection of circPHKA2 in stroke. Furthermore, this protection was partially but distinctively abrogated by inhibiting SOD2 or facilitating miR-574-5p. Molecularly, SOD2 downregulation and miR-574-5p upregulation were paralleled with circPHKA2 downregulation in AIS patients' blood; moreover, there was a linear correlation between SOD2 and circPHKA2 expression in these blood samples. Coupled with direct interactions between miR-574-5p and circPHKA2 or SOD2, these data together revealed a circPHKA2-miR-574-5p-SOD2 ceRNA axis in stroke-induced neurovascular damage.

The characteristics of miR-574-5p association with target genes were suggested to be capable of diagnostic biomarkers in stroke [17]. miR-574-5p-SOD2 interaction was discovered by us in cerebral IS; however, the expression of SOD2 in ischemic lesion tissues was not further detected in this study. Instead, we concentrated on its expression and activity in circulating bloods and OGD-induced AIS model in HBMEC.

Oxidative stress played fundamental role in the pathogenesis of ischemic brain injury, and SOD2 gene polymorphism might contribute to individual variability in oxidative stress status [12, 23]. SOD2 and glutathione were critical for the cellular antioxidant defense in neurodegenerative diseases including cerebral ischemia [24]. Thus, targeting mitochondrial oxidative, focusing on SOD2, might be an effective approach to alleviate ischemic injury. SOD2 expression and activity could be regulated by circPHKA2-miR-574-5p axis in this study, as well as proinflammatory cytokines and ROS overproduction [16]. Besides, SOD2 overexpression could block oxidative stress, ER stress, and apoptosis [25], and our data showed that SOD2 blockage was contributing to those detrimental cell responses after OGD. Angiogenesis participated in the recovery from IS, and SOD2 overexpression in mice reduced vascular endothelial cell death after ischemia and reperfusion [14]. *In vitro*, we noticed that higher SOD2 was one of the consequences of circPHKA2 reexpression in OGD-subjected HBMEC during the salvation of tube formation, proliferation, and migration. This study might be the first clear and convincing evidence that SOD2 upregulation might be conductive to neuroprotection by enhancing vascular endothelial cell proliferation, migration, and neovascularization.

In conclusion, we demonstrated the abnormal downregulation of circPHKA2 in blood of AIS patients and the suppression of circPHKA2 reexpression in OGD-induced HBMEC. Moreover, circPHKA2 protected HBMEC from OGD-induced neurovascular injuries by controlling SOD2 via sponging miR-574-5p. This study indicated that the circPHKA2-miR-574-5p-SOD2 ceRNA axis might be a novel antioxidant-based neurovascular protective strategy in IS. Our research might be the first strong evidence describing the cellular functions of circPHKA2.

## Figures and Tables

**Table 1:** Clinical features of AIS patients and control subjects.

	AIS (*n* = 32)	Control (*n* = 21)	*P* value
Males/females	10/11	17/15	0.695
Age (years)	66.35 ± 2.91	64.23 ± 2.42	0.150
Body mass index (kg/m^2^)	25.12 ± 0.71	25.51 ± 0.82	0.072
Smokers % (*n*)	43.75 (14)	38.10 (8)	0.683
Hypertension % (*n*)	59.37 (19)	33.33 (7)	0.064
Diabetes mellitus % (*n*)	28.13 (9)	19.05 (4)	0.453
Total cholesterol (mmol/L)	4.25 ± 0.51	4.12 ± 0.25	0.284
Triglyceride (mmol/L)	1.55 ± 0.82	1.43 ± 0.71	0.927
HDL-C (mmol/L)	1.02 ± 0.48	1.15 ± 0.30	0.274
LDL-C (mmol/L)	2.51 ± 0.94	2.11 ± 0.85	0.122

Note: AIS: acute ischemic stroke; HDL-C: high-density lipoprotein cholesterol; LDL-C: low-density lipoprotein cholesterol. Data were represented as mean ± standard deviation or percentage (number) for category variables. *P*: AIS *vs*. control.

**Table 2:** The list of qPCR primers.

Name	Sequence (5′-3′)
circPHKA2 divergent primers	TCCTCCAAGTCTGCGAATCC AGATGTTATCCCGCACCCAG (82 bp)
circPHKA2 convergent primers	GTGCCTGAACTCTACGCTGT GTACAAGGATTGGCCCCACA (117 bp)
PHKA2 primers	CTCCAAGTCTGCGAGGTCC TGATGTCCTCCCGAGTCTGA (72 bp)
miR-574-5p primers	GAGTGTGTGTGTGTGAG GAACATGTCTGCGTATCTC (75 nt)
SOD2 primers	AAAGGGGAGTTGCTGGAAGC TGAGCCTTGGACACCAACAG (96 bp)
GAPDH convergent primers	GACAGTCAGCCGCATCTTCT GCGCCCAATACGACCAAATC (104 bp)
GAPDH divergent primers	CCAAGGAGTAAATGGGGAAGG ACCAGAGTTAAAAGCAGCCC (89 bp)
U6 primers	CTCGCTTCGGCAGCACATATACTA ACGAATTTGCGTGTCATCCTTGC (78 nt)

## Data Availability

No data were used to support this study.
